# Optimization and Evaluation of a Novel Size Based Circulating Tumor Cell Isolation System

**DOI:** 10.1371/journal.pone.0138032

**Published:** 2015-09-23

**Authors:** Lei Xu, Xueying Mao, Ahmet Imrali, Ferrial Syed, Katherine Mutsvangwa, Daniel Berney, Paul Cathcart, John Hines, Jonathan Shamash, Yong-Jie Lu

**Affiliations:** 1 Centre for Molecular Oncology, Barts Cancer Institute, Queen Mary University of London, London, United Kingdom; 2 Department of Urology, Zhongshan Hospital, Fudan University, Shanghai, China; 3 Department of Medical Oncology, Barts Health NHS, London, United Kingdom; 4 Centre for Experimental Medicine, Barts Cancer Institute, Queen Mary University of London, London, United Kingdom; 5 Department of Urology, University College Hospital NHS, London, United Kingdom; 6 Department of Urology, Barts Health NHS, London, United Kingdom; Chang Gung University, TAIWAN

## Abstract

Isolation of circulating tumor cells (CTCs) from peripheral blood has the potential to provide a far easier “liquid biopsy” than tumor tissue biopsies, to monitor tumor cell populations during disease progression and in response to therapies. Many CTC isolation technologies have been developed. We optimized the Parsortix system, an epitope independent, size and compressibility-based platform for CTCs isolation, making it possible to harvest CTCs at the speed and sample volume comparable to standard CellSearch system. We captured more than half of cancer cells from different cancer cell lines spiked in blood samples from healthy donors using this system. Cell loss during immunostaining of cells transferred and fixed on the slides is a major problem for analyzing rare cell samples. We developed a novel cell transfer and fixation method to retain >90% of cells on the slide after the immunofluorescence process without affecting signal strength and specificity. Using this optimized method, we evaluated the Parsortix system for CTC harvest in prostate cancer patients in comparison to immunobead based CTC isolation systems IsoFlux and CellSearch. We harvested a similar number (p = 0.33) of cytokeratin (CK) positive CTCs using Parsortix and IsoFlux from 7.5 mL blood samples of 10 prostate cancer patients (an average of 33.8 and 37.6 respectively). The purity of the CTCs harvested by Parsortix at 3.1% was significantly higher than IsoFlux at 1.0% (p = 0.02). Parsortix harvested significantly more CK positive CTCs than CellSearch (p = 0.04) in seven prostate cancer patient samples, where both systems were utilized (an average of 32.1 and 10.1 respectively). We also captured CTC clusters using Parsortix. Using four-color immunofluorescence we found that 85.8% of PC3 cells expressed EpCAM, 91.7% expressed CK and 2.5% cells lacked both epithelial markers. Interestingly, 95.6% of PC3 cells expressed Vimentin, including those cells that lacked both epithelial marker expression, indicating epithelial-to-mesenchymal transition. CK-positive/Vimentin-positive/CD45-negative, and CK-negative/Vimentin-positive/CD45-negative cells were also observed in four of five prostate cancer patients but rarely in three healthy controls, indicating that Parsortix harvests CTCs with both epithelial and mesenchymal features. We also demonstrated using PC3 and DU145 spiking experiment that Parsortix harvested cells were viable for cell culture.

## Introduction

The molecular alterations in cancer cells change and evolve during cancer progression and in response to therapeutics. Understanding the changes in tumor cell populations during disease progression and in response to therapies requires frequent cancer cell sampling, which is practically difficult using invasive tumor tissue biopsies. It is now known that a large number of tumor cells can be released into the blood circulation long before tumor metastasis occurs and even at very early stage of cancer development [1, 2]. One or more circulating tumor cells (CTCs) were reported to be detected in 100% of seven patients with early-stage prostate cancer [3], 19.6% of 692 node-negative breast cancer [4] and 24% of stage 1–3 breast cancer patients [5]. Those CTCs not only offer the potential to study the mechanism of cancer metastasis, but also allow real-time monitoring of cancer progression and prediction of therapy response in a minimally-invasive manner, which is referred to as liquid biopsy [6]. CTCs have been included in >400 clinical trials to evaluate their potential as a diagnostic/prognostic biomarker, mainly based on CTC enumeration [7]. Extending this to genetic analysis of CTCs will further reveal the molecular mechanisms and intratumoral heterogeneity at individual cancer cell level to inform on cancer progression and response/resistance to therapies. It has also been reported that CTCs associated with cancer metastasis acquired more genomic alterations than those detected in primary tumors [8–10]. Therefore, genetic analysis of CTCs may also reveal more molecular alterations associated to the deadly metastatic tumors than data generated from surgical tissue samples of the primary tumors.

Several CTC capture and/or isolation systems have been developed. The bead-based epithelial cell adhesion molecule (EpCAM) antibody CTC capturing system is most widely used and one of them, CellSearch, the only Food and Drug Administration (FDA) approved device for CTC analysis (enumeration), is considered the current “gold standard” [11]. However, the performance and applicability of this system is receiving more and more challenge, especially regarding the capture rate and purity [12]. Several other emerging systems, such as the MagSweeper system [13], IsoFlux system [14] and Microvortex chip [15], which are based on a similar principle but with improved technologies, were reported to have both a higher sensitivity and purity. However, the EpCAM-based approach of CTC isolation is recognized to have a critical limitation. During cancer metastasis, epithelial-to-mesenchymal transition (EMT) occurs to increase the invasion capability of cancer cells [16]. This process leads to down-regulation of epithelial markers, such as EpCAM and up-regulation of mesenchymal markers. New CTC isolation strategies based on cell properties other than cell membrane expressed proteins, such as cell size and density, may help combat this problem. Several size-based systems have been developed [6]. The Parsortix system is one such cell size-based system utilizing microfluidic technology and is a CE marked *in vitro* diagnostic device in the EU.

Prostate cancer is the most common noncutaneous cancer and the second most frequent cause of cancer-related death in European men [17]. Prostate specific antigen (PSA) is a widely used biomarker for detecting and monitoring this disease. Although, so far, no available alternative biomarkers can replace it, its limitations in stratifying patients with aggressive tumors and in reflecting therapy response in castration resistance stage presents a strong medical need for researchers to discover a better or at least a supplementary biomarker to improve clinical management for prostate cancer patients. Detection of prostate cancer antigen 3 (PCA3) in urine is another emerging biomarker for prostate cancer. PCA3 is reported to be localized to prostatic tissue and found in 95% of prostate cancer and prostate metastasis specimens [18]. Moreover, it can be detected from specimens containing as little as 10% cancer [19]. It is mainly applied in the assistance of diagnosis, in particular, to determine the necessity of a second biopsy. As a “liquid biopsy”, CTCs store more genetic and cellular information than cell-free circulating nucleic acid and proteins. Here we used prostate cancer as a cancer model to optimize and evaluate the Parsortix size and compressibility-based system (Parsortix Cell Separation, ANGLE plc, UK) for CTC isolation, and compared its efficiency with IsoFlux (Fluxion Biosciences Inc, South San Francisco, CA) and CellSearch systems (Veridex, Warran, NJ). We optimized the Parsortix system, making it possible to process samples at a speed and sample volume comparable to the standard CellSearch system for clinical sample analysis and demonstrated that it can harvest CTCs not only with epithelial features, but also in the process of or completed EMT. The harvested CTCs were easily utilized for downstream molecular analysis and were viable for cell culture.

## Materials and Methods

### The Parsortix system

**Fig 1:**
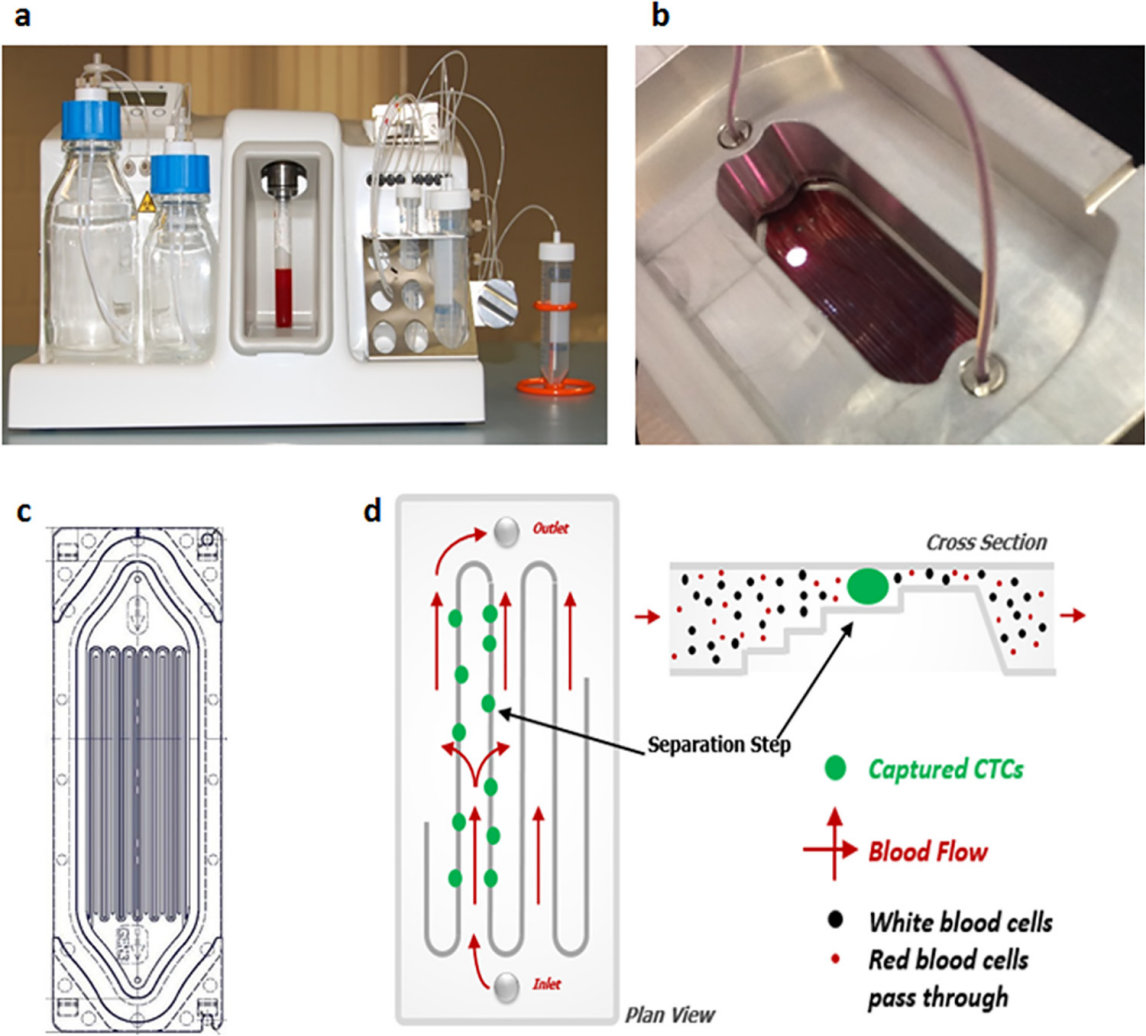
Parsortix system overview and cassette design. (A) Overview of Parsortix system. (B) Overview of the clamp holding the cassette where the blood passes through. (C) A diagram of the disposable isolation cassette. (D) Isolation principle inside the cassette. Blood is forced along a series of channels and to flow through a 10 μm patented step which separates particles on the basis of size and compressibility.

The Parsortix system (**Fig 1A and 1B**) utilizes microfluidic based particle separation technology, with a disposable cassette (**Fig 1C**) containing a separation structure comprising a series of steps across which cells are forced to pass, leading them to a terminal gap of 10 μm. Most blood cells pass across the steps and through the terminal gap and cells whose size and rigidity prevent them from passing through the step structure (e.g. CTCs) are retained in the cassette (**Fig 1D**).

### Cell lines

Two human prostate cancer cell lines, PC3 and DU145, and a human breast cancer cell line, MCF-7, were used as spiked cancer cells to simulate cancer samples as a validation test. All the cell lines are from ATCC.

### Patient and control samples

Blood samples collected with patient consent were obtained from 15 metastatic prostate cancer patients and four newly diagnosed localized prostate cancer patients prior to any treatment at St Bartholomew's Hospital, Barts Health NHS, London, UK. 17 cancer-free participants were recruited for normal controls or spiking experiments with signed Ethics Committee approved consent form. Of those cancer patient samples, eight metastatic and two localized cases were analyzed in parallel with the IsoFlux system, and seven metastatic cases were analyzed in parallel with CellSearch. Three samples were tested on all the three CTC isolation systems (**Table 1**). The remaining blood samples from three metastatic and two untreated localized prostate cancer cases (**Table 1**) were used for CTC isolation only by the Parsortix system to investigate potential of captured EMT cells. All cancer cases were diagnosed by tissue biopsy. 12 samples from healthy individuals were used for cell line spiking experiments and five were used as normal blood controls (two as negative controls in system comparison, and the other three as negative controls in EMT investigation). Blood samples were drawn into EDTA Vacutainer tubes (Becton Dickinson and Company, Polymouth, UK) and acquired at the middle of phlebotomy after the collection for routine clinical blood test to avoid contamination with epithelial cells from the skin. All blood samples were stored at room temperature and processed within 24 hours of blood draw.

**Table 1:** Clinical information and disease status of prostate cancer patients.

Case ID	Age	GS on initial biopsy	Time from biopsy (month)	PSA on sample collection (ng/mL)	Metastases status	CRPC Y/N	Isolation Platform
PC2	80	3+5	64.8	206	M.B.; RP Lym	Y	Par/Iso
PC5	61	4+5	34.2	35	Two bones	Y	Par/Iso/Cel
PC15b	91	3+4	57.0	478	M.B.	Y	Par/Iso/Cel
PC16	82	3+4	175.1	401	M.B.; RP Lym	Y	Par/Iso
PC17	68	4+5	126.5	4421	M.B.; RP Lym	Y	Par/Iso
PC19	74	5+4	107.6	255	RP,P Lym	Y	Par/Iso
PC7b	61	4+5	66.1	399	RP Lym	Y	Par/Iso/Cel
PC20	77	4+3	113.8	834	M.B.	Y	Par/Iso
PC28	58	5+4	1.3	25	None	N	Par/Iso
PC37	76	3+3	2.7	10	None	N	Par/Iso
PC6	85	4+3	23.9	36.9	M.B.; P Lym	Y	Par/Cel
PC2c	80	3+5	66.4	160	RP Lym	Y	Par/Cel
PC22b	83	3+3	24.2	6000	M.B.	Y	Par/Cel
PC41	70	4+5	11.1	39	Three bones	Y	Par/Cel
PC32b	70	5+4	34.0	830	M.B	Y	Par
PC36	81	4+5	73.7	260	M.B.; RP Lym	Y	Par
PC39	82	3+4	65.8	12	Single Bone	N	Par
PC40	59	4+4	1.0	22	None	N	Par
PC46	57	4+3	1.0	13	None	N	Par

GS: Gleason score; M.B.: multiple bone metastases; RP: retroperitoneal; P: pelvic; Lym: lymph nodes metastasis. Par: Parsortix; Iso: IsoFlux; Cel: CellSearch.

### Preparation of Spiked Samples

PC3, DU145 or MCF-7 cells were pre-labeled by CellTracker Green (Invitrogen, UK) according to manufacturer’s instructions. Labeled cells were confirmed under fluorescence microscope before trypsinization. Cells suspended in growth medium were diluted to a concentration of 5000 cells/mL. Triplicate 10 μL of the diluted solution were then dropped on slides for manual counting to confirm/adjust to 50 cells per drop. A number of pre-labeled cancer cells were then spiked into 7.5 mL of whole blood from healthy donors.

### Measurement of Cell Size

The diameters of PC3/DU145/MCF-7 cells and human lymphocytes in suspension were measured by Vi-Cell XR 2.03 (Beckman Coulter, Inc.) according to manufacturer’s instructions before spiking.

### Parsortix Sample Preparation and CTC Isolation

Two approaches were tested to isolate cells by Parsortix. 1. Dilute whole blood in PBS by 1:1 ratio and load the sample onto machine. 2. Buffy-coat recovery approach. Buffy-coat recovery approach requires 7mL out of 7.5mL of blood to 50 mL LeucoSep tubes (Greiner Bio-One, Germany) with 15 mL of Ficoll-Paque Plus (GE Healthcare, Sweden) to recover peripheral blood mononuclear cell (PBMC) fraction at 800g for 15 min with break off at room temperature. PBMC fraction along with the plasma above the frit of LeucoSep tube were removed to a new 50 mL conical tube and pelleted at 300g for 10 min at room temperature. The pellet was re-suspended in 4.5 mL of isolation buffer (PBS containing 1% BSA and 2mM EDTA) and added back to the remaining 0.5 mL of whole blood to load onto the Parsortix for cancer cell isolation. Once samples were loaded, cells were separated according to the pre-set program.

### CTC recovery rate calculation using spiked samples

CTC recovery rate was calculated as capture rate and harvest rate. Capture rate was calculated as number of spiked cells trapped in cassette compared to the total spiked cells. Harvest rate was based on number of spiked cells eluted from the system on to a slide.

### Harvesting Parsortix isolated CTCs onto a glass slide

The harvest program of Parsortix performs 20 short pulses to dislodge captured cells. 200 μL of buffer containing cells is eluted out of the cassette and ready for analysis. We tested several cell transfer and fixation methods to maximize the number of cells transferred onto the glass slides for downstream CTC analysis. Two cell transfer methods were tested. Firstly, Cytospin was used for the transfer. Eluted cell solution was loaded into chamber of Cytospin3 (Shandon, USA), which had a pre-spin to wet the filter card with 20 μL of PBS and then spin 200 μL of harvested cells at 500RPM for 6 min. We also tried to drop the eluted cell solution directly onto slide and air dry. We initially tested three fixation methods: 4% paraformaldehyde at room temperature for 10 min, 100% acetone on ice for 20 min and 100% methanol on ice for 20 min. To further optimize the Acetone fixation, we tested the following modified fixation methods: 1. PBS-70% Acetone: cells were suspended in PBS and air dried on slides, followed by fixation with 70% acetone in distilled water on ice for 20 min; 2. KCl-70% Acetone: cells were suspended in 0.075M KCl and air dried on slides, followed by fixation with 70% acetone in distilled water on ice for 20 min; and 3. KCl-Acetone: cell were suspended in 0.075M KCl and air dried on slides, followed by fixation with 100% acetone on ice for 20 min. All slides were coated with ploy-L-lysin (Sigma-Aldrich, Life Science, USA) before use. Immunofluorescene analysis (for EpCAM and Vimentin) with DAPI nuclear staining was done after different fixation to compare the efficiency of cell retention on slides—percentage of cells kept on slide after immunostaining.

### Isolation by IsoFlux

PBMC pellet from 7.5 mL of whole blood was obtained as above, re-suspended in 1 mL of binding buffer (CTC Enrichment Kit; Fluxion Biosciences Inc). Samples were mixed with anti-EpCAM antibodies pre-conjugated immunomagnetic beads (CTC Enrichment Kit; Fluxion Biosciences Inc.) and incubated for 2 h at 4°C with passive mixing on a rotator. Following the beads coupling step, samples were loaded into IsoFlux and separated according to manufacturer’s instruction. The captured cells were recovered from the isolation zone disk with 20 μL of binding buffer, transferred into a microfuge tube and fixed by 2% paraformaldehyde for 10 min at room temperature.

### Isolation by CellSearch

Procedures were performed as standard according to manufacturer’s instruction. In brief, 7.5 mL of blood mixed with 6.5 mL of dilution buffer were centrifuged and transferred into the CellTracks AutoPrep system. The remaining procedures were done automatically by the instrument. The CellSearch Epithelial Cell kit (Veridex, Warren, NJ) containing ferrofluid particles coated with anti-EpCAM antibodies were used.

### Immunofluorescence staining

For spiked cells or CTCs to be analyzed on slides, three or four color Immunofluorescence staining was used to identify different populations of cells.

Three-color Immunofluorescence: After blocking by 10% normal donkey serum for 10 min, cells were incubated with rabbit polyclonal anti-human CD45 antibody for 30 min and followed by Alexa Fluor546 donkey anti-rabbit antibody (Life technology, USA) for 20 min. Cells were permeabilized with 0.1% Triton X-100 for 5 min, stained with FITC conjugated anti-cytokeratin (CK) (clone CK3-6H5, Miltenyi Biotec, Germany) for 40 min and mounted in SlowFade gold antifade mountant with DAPI (Life technologies).

Four-color Immunofluorescence: After blocking by 10% normal donkey serum for 10 min, cells were incubated with mouse monoclonal anti-human CD45 (Santa Cruz) and rabbit monoclonal anti-human Vimentin (Epitomics) for 30 min followed by Alexa Fluor647 donkey anti-mouse antibody and Alexa Fluor546 donkey anti-rabbit antibody for 20 min. Then cells were permeabilized with 0.1% Triton X-100 for 5 min, stained with FITC conjugated anti-CK for 40 min, and mounted in SlowFade gold antifade mountant with DAPI.

### CTC enumeration

Pre-labeled spiked cells were counted directly in the cassette under Axioplan fluorescent microscope (Carl Zeiss). Slides with immunofluorescence staining were scanned using Ariol image analysis system (Leica Microsystems (Gateshead) Ltd, UK), equipped with an Olympus BX61 microscope. In three-color immunofluorescence result, cells with CK positive /CD45 negative /DAPI positive and morphologically intact nucleus were counted as CTCs, while other cells were also marked. Number of CTCs was recorded and purity was calculated by the ratio of number of CTCs to the total cells on slides. In four-color staining study, cells with CK positive/Vimentin negative/CD45 negative, CK positive/Vimentin positive/CD45 negative, and CK negative/Vimentin positive/CD45 negative were counted.

### Viability of Parsortix harvested cancer cells

#### Cell viability analysis

200 CellTracker Green pre-labeled PC3 and DU145 cells were spiked into 7.5 mL of whole blood and isolated and harvested by Parsortix. Trypan Blue (Sigma) exclusion was used to determine viability of the harvested cells. Cells with no trypan blue staining were considered viable and those stained with blue dye were considered as non-viable. Three repeats were performed to calculate the mean viability.

#### Cell proliferation ability in culture

200 μL of harvested cells were prepared as above and collected in Fisherbrand siliconized low-Retention microcentrifuge tube (Fisher Scientific), spun at 2000 RPM for 5 min and re-suspended in 200 μL of growth medium (DMEM (Sigma-Aldrich) with 10% fetal calf serum (Gibco, Life technologies) and 1 × antibiotic-antimycotic (Sigma-Aldrich)). Cells were then transferred into a well of a 24-well plate and topped up with 1 mL growth medium for culture. Growth medium was changed on day 2 and then every 4 days. Cells were observed daily under inverted microscopes.

### Statistical analysis

Data from replicated experiments were reported as means ± SD. Difference in means was compared by t-test between two groups and by one-way ANOVA among three or more groups. Difference in the number of CTCs harvested by different isolation platform was compared by Wilcoxon signed-rank test.

## Results

### 1.Increasing Parsortix sample process speed and blood volume capacity by buffy coat mononuclear cell separation

At the time of this study, it was not possible to process more than 3 mL of whole blood using the Parsortix system, and alternative pre-processing methods were required. 1:1 dilution in PBS and buffy coat recovery were tested. As both from Parsortix manufacturer’s and our tests, the re-suspension of a very high number of PBMC cells in relatively small volume of PBS could clog the CTC isolation system, we improved the PBS buffer by adding 1% BSA and 2mM EDTA. Using this buffer, we compared 1:1 dilution and buffy-coat recovery methods for their capacity to process 7.5 mL of blood samples. The efficiency of cancer cell harvest was also compared by spiking 100 CellTracker Green pre-labeled PC3 cells into the normal blood samples to assess cancer cell capture rates in cassettes and harvest rates on slides. Four repeated tests were performed and the buffy-coat recovery method processed the sample significantly faster (p < 0.0001) than 1:1 dilution method (**Table 2**). Although the in cassette capture rate of 1:1 dilution looked slightly higher than buffy-coat recovery methods (p = 0.38), the final harvest rates were similar from the two methods (p = 0.77) (**Table 2**). The differences in both capture and harvest rate had no statistical significance. Following this comparison, we applied buffy-coat recovery methods for further experiments using the Parsortix system.

**Table 2:** Comparison of three different samples pre-treatments for Parsortix.

	Blood volume (mL)	Mean separation time (minute)	Mean capture Rate (%)	Mean harvest rate (%)
1:1 dilution	7.5	188.5 ± 3.3**	62.5±3.9	43.5±7.0
Buffy-coat recovery	7.5	43.3 ± 1.3**	59.0±6.2	44.8±4.5
p value		<0.0001	0.38	0.77

**n = 4.

### 2.Capture rate of different cell lines spiked in blood samples

**Fig 2:**
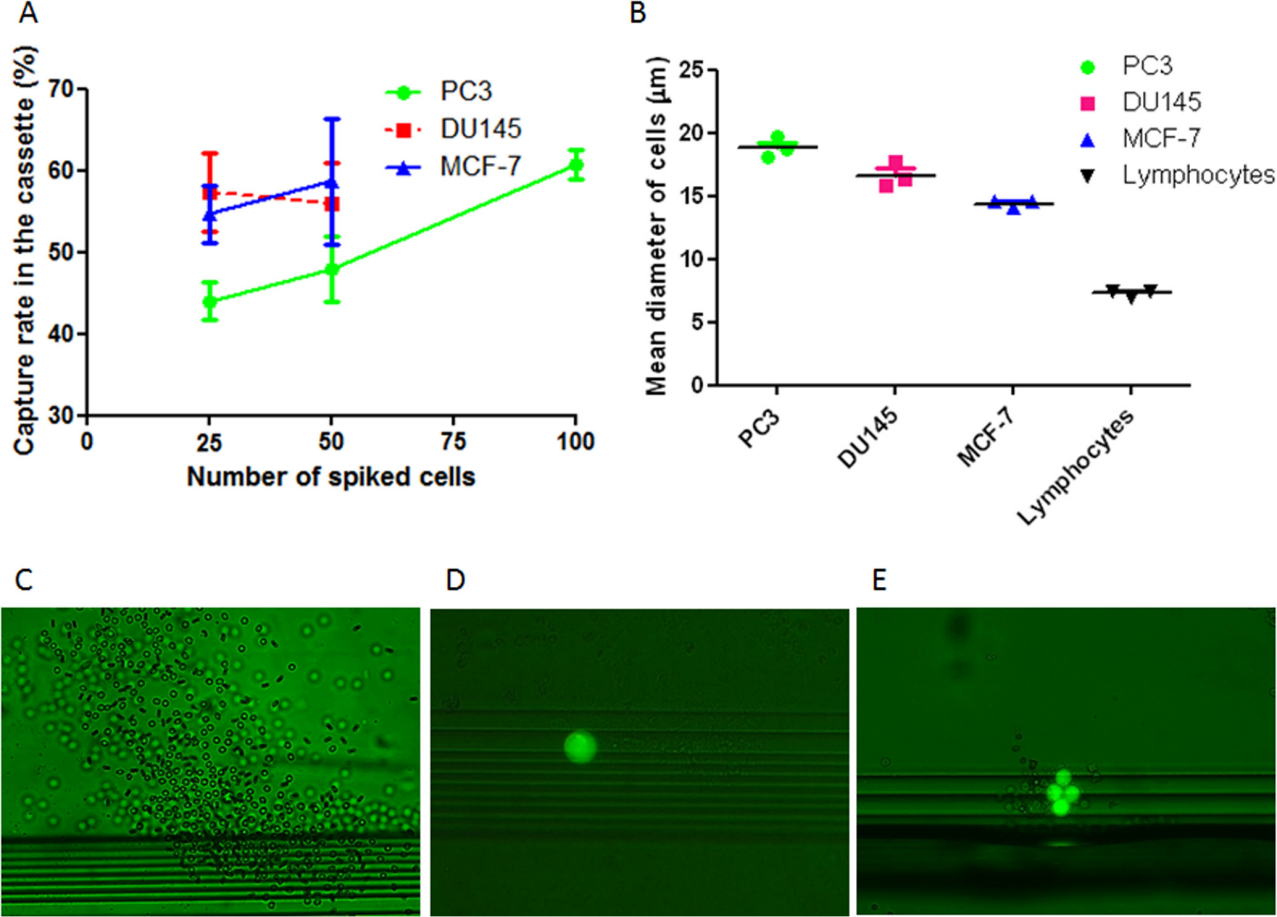
Spiked cancer cell lines test for capture rates. (A) Mean capture rate for spiked PC3, DU145 and MCF-7 after isolation. (B) Three repeated measurements for the mean diameter of PC3, DU145, MCF-7 and normal human lymphocytes. (C-E) representative examples of (C) smaller lymphocytes in the process of passing through the gap and exiting Parsortix cassette to the waste, magnification 100X); (D) captured pre-labeled single spiked cell in the stepped gap, magnification 200X; and (E) captured pre-labeled cluster of spiked cells in the stepped gap, magnification 200X.

The mean capture rate of PC3 cells spiked into normal blood was 54.4% ± 9.1% (n = 14) with capture rate for 25 (n = 3), 50 (n = 3) and 100 (n = 8) spiked cells at 44.0% ± 4.0%, 48.0% ± 6.9% and 60.8% ± 5.2% respectively (**Fig 2A**). The mean capture rates of 25 (n = 3) and 50 (n = 3) spiked DU145 cells were 57.3% ± 8.3% and 56.0% ± 8.7%, respectively (**Fig 2A**). The mean capture rates of 25 (n = 3) and 50 (n = 3) spiked MCF-7 cells were 54.7% ± 6.1% and 58.7% ± 13.3%, respectively (**Fig 2A**). The mean diameters of PC3, DU145, MCF-7 and normal lymphocytes measured by Vi-Cell XR 2.03 were 18.8 ± 3.8, 16.8 ± 3.7, 14.4 ± 3.2 and 7.3 ± 1.9 μm, respectively (**Fig 2B**). Examples of lymphocytes passing through the isolation gaps to the waste collection during the process were shown in **Fig 2C** and captured pre-labeled spiked cancer cells under fluorescence microscope were shown in **Fig 2D and 2E**.

### 3.Optimization of methods to harvest isolated CTCs onto glass slide for molecular analysis

As CTCs are extremely rare and hence only a small number of target cells are harvested, efficient transfer of cells in 200 μL of solution on to a slide and fixation for immunofluorescence staining is critical to enable the analysis of as many cells as possible without affecting immunostaining signal specificity and strength. We initially used PC3 to test two sample transfer methods: Cytospin and directly loading, combining with three most commonly used fixation methods, 4% paraformaldehyde, 100% acetone and 100% methanol. Immunofluorescence of EpCAM and Vimentin was done to compare the efficiency between different methods. 20 μL of PC3 cell suspension for direct loading was initially used to shorten the air-dry time.

**Fig 3:**
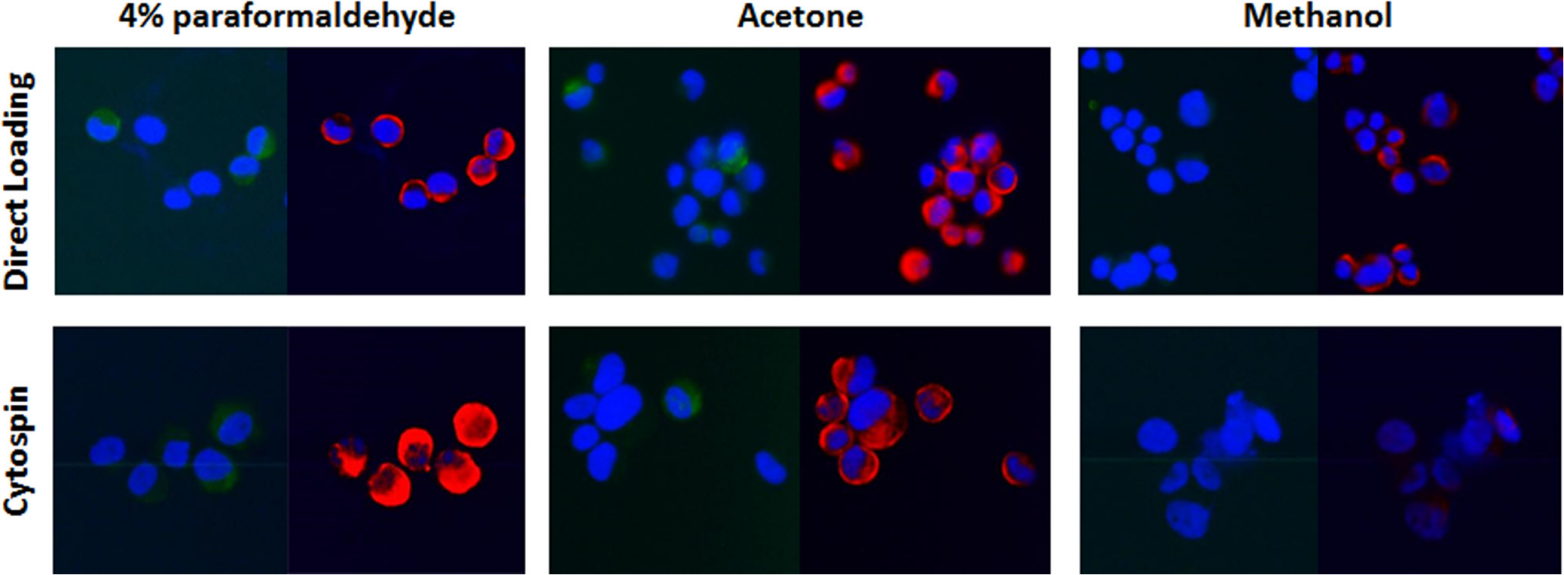
Immunostaining of EpCAM and Vimentin for cultured cells after different cell transfer and fixation methods. EpCAM was shown as green signals and Vimentin was shown as red signals. Exposure time and magnification of images were the same for each method. Based on the same fixation, EpCAM signals by direct transfer were stronger than those by Cytospin. In addition, the morphology of cells was flattened after cytospin. Based on the same transfer method, paraformaldehyde provided the brightest signals and methanol provided the weakest and uneven signals.

Acetone fixation helped keep most cells (**Table 3**) and provided similar signal specificity and strength compared to paraformaldehyde (**Fig 3**). Paraformaldehyde provided brightest signals (**Fig 3**), but retention rates (the proportion of cells kept on the slide after immunostaining out of the number of cells used to load on to the slide) were not stable (**Table 3**). While methanol also kept most cells, immunofluorescence signals were very weak and uneven, especially for the membrane protein (**Fig 3**). Cells transferred on to a slide by Cytospin can be well-retained with all fixation after immunostaining (**Table 3**). However, the Cytospin process caused the loss of half the cells and cell morphology was affected (**Fig 3**).

**Table 3:** Cell retention rates for different transfer and fixation combination using 20 μL cell suspension.

	Direct loading	Cytospin
4% paraformaldehyde	52.3 ± 18.1%	46.5 ± 4.9%
Acetone	91.3 ± 3.0%	46.8 ± 4.9%
p value	p<0.0001	p = 0.40

**Fig 4:**
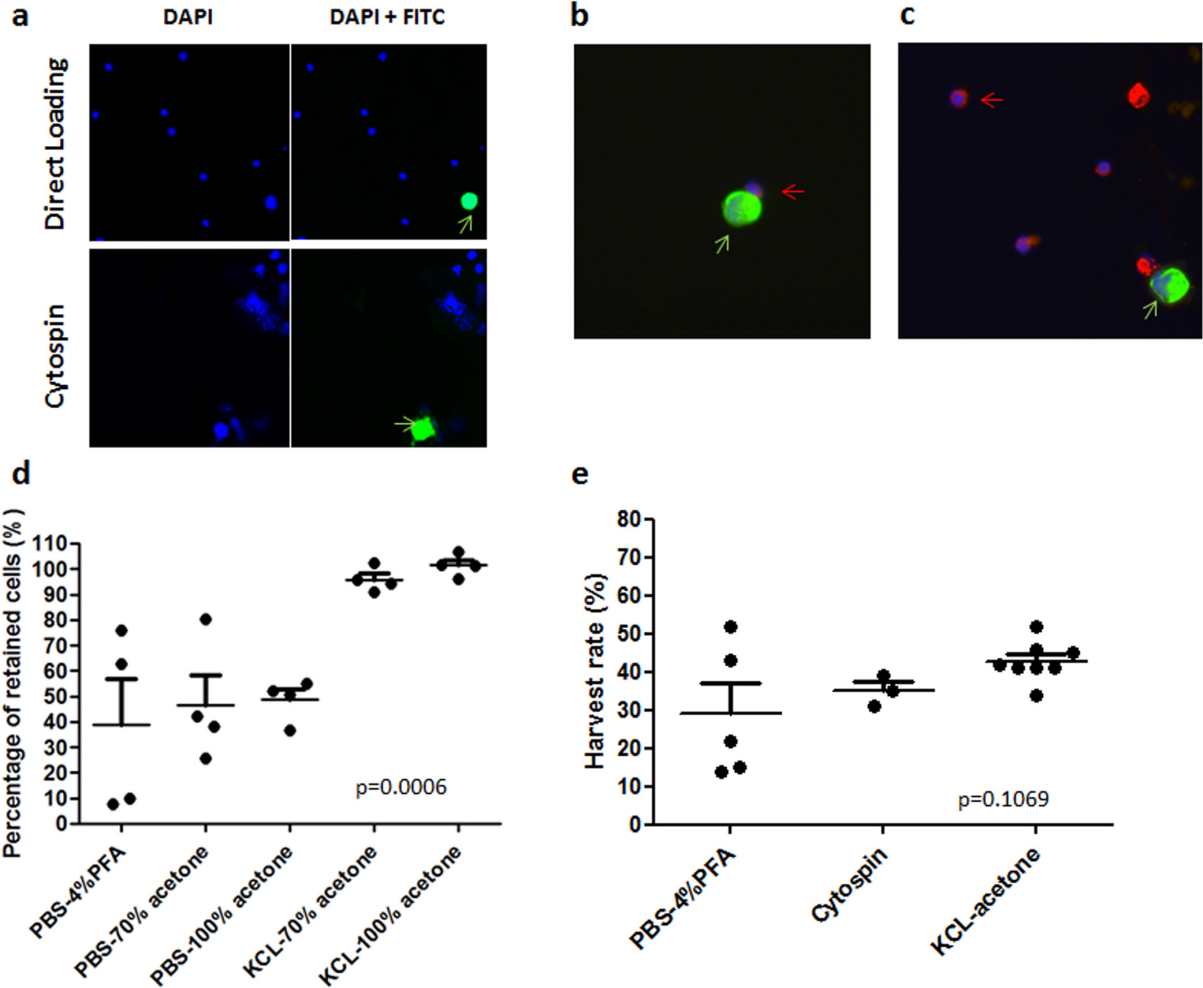
Optimization of cell transfer and fixation for immunofluorescence analysis using spiked samples. (A) Representative images to show a CellTracker Green pre-labeled PC3 cell with damaged morphology (green arrow) after Cytospin (lower panel), compared with a round pre-labeled PC3 cell (green arrow) in direct cell loading method (upper panel). (B) and (C) Using both (B) PBS-4% paraformaldehyde and (C) KCl-acetone cell transfer and fixation methods, CK expression (green signals) on PC3 cells (green arrows) and CD45 expression (red signals) on lymphocytes (red arrows) were clearly detected with strong and specific fluorescence signals. (D) Percentage of cells retained after immunofluorescence staining process for cells with different re-suspension solutions and fixations. Both 70% and pure acetone fixation in combination with KCl re-suspension of cells achieved a retention rate of more than 90%. (E) Harvest rates of spiked PC3 cells using different cell transfer and fixation methods. KCl-acetone had the highest rate, Cytospin had a lower but more stable rate and PBS-4% paraformaldehyde had the lowest and most unstable rate.

We further tested the IF signal for 100 pre-labeled PC3 cells spiked in 7.5mL of blood from healthy donors using direct loading followed by 100% acetone or 4% paraformaldehyde, and Cytospin followed by fixation with 4% paraformaldehyde. By triplicate experiments, the harvest rates were 21%, 32% and 12% for acetone fixation, 15%, 52% and 43% for paraformaldehyde fixation with directly loaded cells, and 35%, 39% and 31% for paraformaldehyde fixation of Cytospin transferred cells. The morphology of PC3 after Cytospin was obviously damaged (**Fig 4A**). The harvest rates for direct loading followed by acetone fixation were surprisingly lower than expected 40–50% and were even lower than by 4% paraformaldehyde fixation. During the air dry, we noticed that a thick layer of salt was built up on the slide when 200 μL of cell solution in PBS was transferred to a small area of the slide, which may affect the fixation. Therefore, we further tested the fixation by adding water to acetone or replacing PBS with 0.075M KCl to re-suspend the cells in order to reduce the crystal salt. Using 200 μL of PC3 cell solution, we tested the harvest rates for 1. Harvest in PBS, fixed by 4% paraformaldehyde, 2. Harvest in PBS, fixed by 70% acetone, 3. Harvest in PBS, fixed by 100% acetone, 4. Harvest in 0.075M KCl, fixed by 70% acetone, and 5. Harvest in 0.075M KCl, fixed by 100% acetone. Harvest in 0.075M KCl and fixed by either 70% or 100% acetone provided the highest retention rate of more than 90% cells (**Fig 4D**). We chose KCl-100% acetone method in comparison to PBS-4% paraformaldehyde for further spiking test (100 non-labeled PC3 spiked in 7.5 mL of blood) with CK and CD45 immunofluorescence staining. Eight repeats for KCl-100% acetone and two repeats for PBS-4% paraformaldehyde showed that the mean recovery rate for KCl-100% acetone was 42.8% (ranging from 34–52%) and the two harvest rates for PBS-4% paraformaldehyde were 14% and 22%. Immunostaining signals after paraformaldehyde or acetone fixation showed a similar signal brightness and pattern for CK on PC3 cells and CD45 on lymphocytes (**Fig 4B and 4C)**. Combined data of all spiked tests using the three different methods were shown in **Fig 4E**, and the mean harvest rates for PBS-4% paraformaldehyde (n = 5), Cytospin-4% paraformaldehyde (n = 3) and KCl-acetone (n = 8) were 29.2% ± 17.3% (p = 0.06, compared to KCl-acetone), 35.0% ± 4.0% (p = 0.045, compared to KCl-acetone) and 42.8% ± 5.2%, respectively, demonstrating that KCl-acetone is the best method to fix cells onto slide for immunofluorescence analysis. When the purity was calculated by the ratio of harvested PC3 to total cells on slide, it was 4.6% ± 1.8% for KCl-acetone with average of 927 cells harvested from 7.5 mL of whole blood.

### 4.Comparison of Parsortix with IsoFlux and CellSearch for CTC harvesting

#### Spiked PC3 cells

Two spiked samples using pre-labeled PC3 cells in 7.5 mL of normal blood were tested for IsoFlux to compare with the Parsortix results. The IsoFlux harvest rates for 20 (n = 1) and 100 (n = 1) spiking cells were 90% and 93%, respectively, which was higher than the harvest rate by Parsortix (42.8%) as described earlier. However, the total numbers of IsoFlux harvested cells counted on slides were 3256 and 3316 respectively, resulting in purities of 0.5% and 2.7% respectively. Therefore, using 100 PC3 spiked blood samples, the purity of IsoFlux harvested cancer cells was lower than that of Parsortix (4.6%).

#### Clinical samples

**Fig 5:**
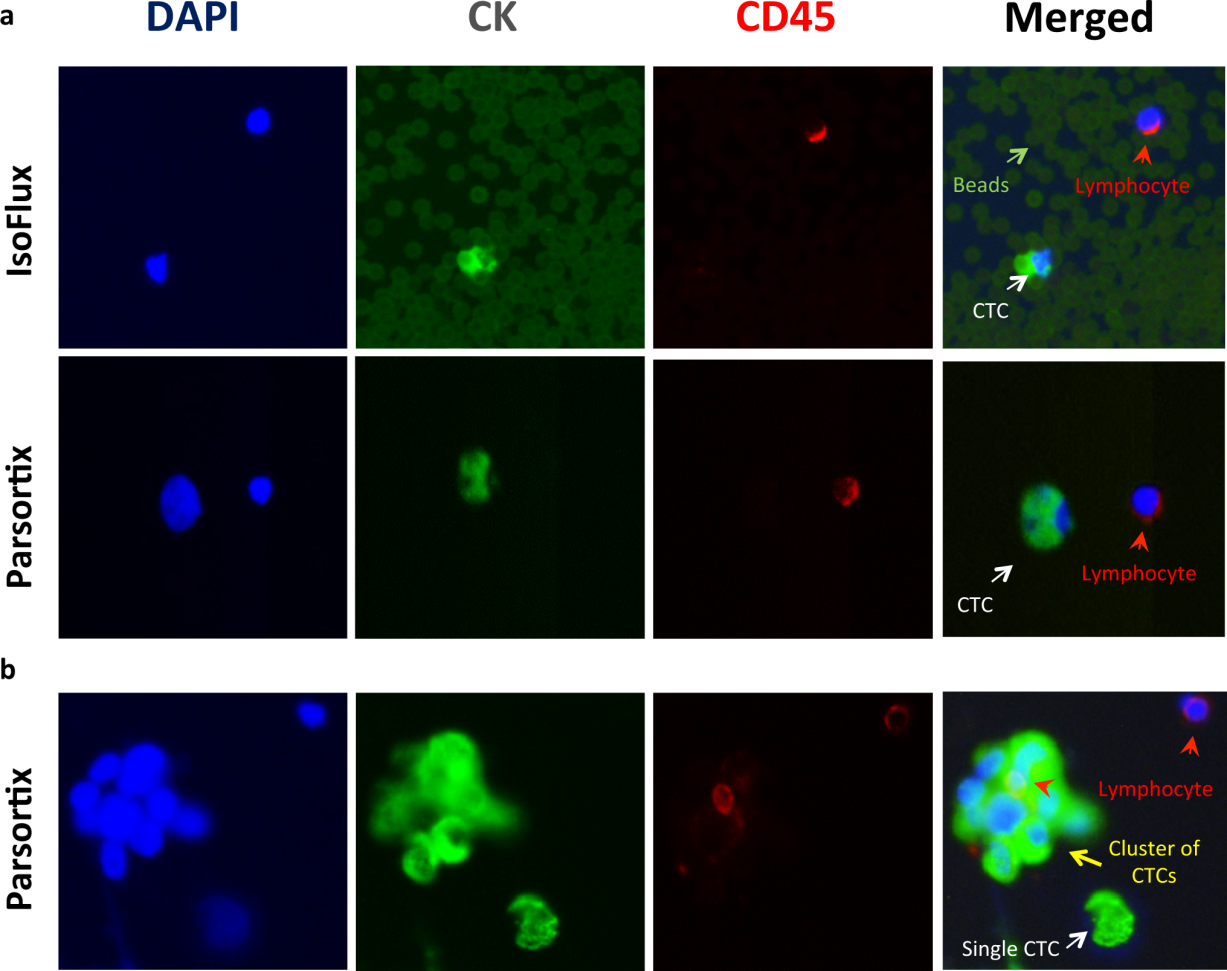
Representative images for the identified CTCs in parallel study. (**A**) Images of single CTCs. The upper row: cells harvested by IsoFlux with beads around cells. The lower row: cells harvested by Parsortix without beads around. (**B**) A cluster of CTCs (yellow arrow), single CTC (white arrow) and surrounding lymphocytes (arrowhead) were observed in samples harvested by Parsortix. Signals for nucleus, cytokeratin and CD45 were presented separately and then merged together. A CTC is defined as a CK positive/CD45 negative, nucleated and morphologically intact cell.

Blood samples (7.5 mL for each system) from 14 patients including two with newly diagnosed untreated localized disease were analyzed by at least two systems. Three samples (PC 5, 15b & 7b) have been analyzed by all the three systems (**Tables 1 and 4**). Immunofluorescence staining detected a small population of CK positive, CD45 negative cells (**Fig 5**), a large population of CK negative, CD45 positive cells (**Fig 5**). In addition there were also small numbers of CK and CD45 both positive or both negative cells. In the two healthy donors, no CK positive, CD45 negative, nucleated and morphologically intact cell was detected using Parsortix and IsoFlux.

**Table 4:** Number of CTCs harvested by different systems in Parallel CTC analysis of prostate cancer patient samples.

Case ID	Parsortix	IsoFlux	CellSearch
PC2	39	50	-
PC5	94	36	2
PC15b	19	22	5
PC16	54	64	-
PC17	7	27	-
PC19	8	33	-
PC7b	23	36	19
PC20	38	76	-
PC28	2	5	-
PC37	54	27	-
PC6	17	-	2
PC2c	43	-	19
PC22b	12	-	22
PC41	17	-	2

-: No data.

**Fig 6:**
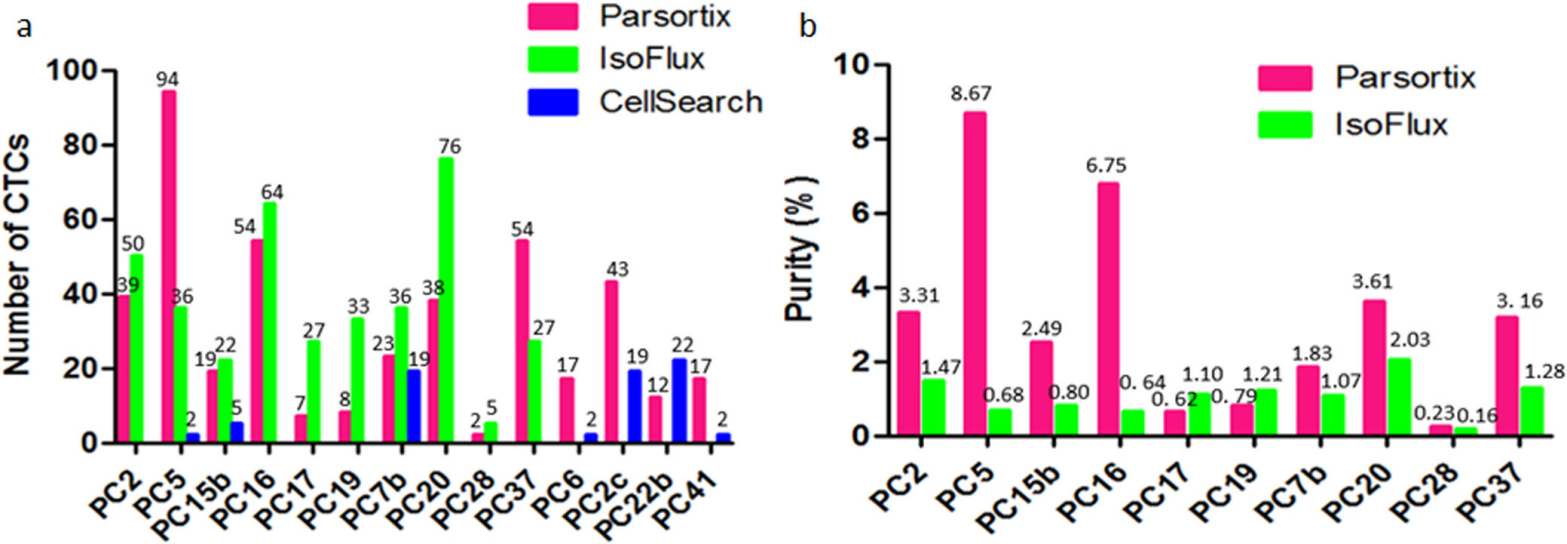
Comparison of the number and purity of harvested CTCs among three platforms. **(A**) Number of harvested CTCs of each patient by different system. (**B**) CTC purity of the harvested sample of each patient by Parsortix and IsoFlux.

Based on the current generally accepted definition of CTCs as CK positive, CD45 negative, nucleated and morphologically intact cells, CellSearch harvested least CTCs among the three platforms. Between Parsortix and IsoFlux, Parsortix harvested more CTCs in one metastatic castration-resistant prostate cancer (CRPC) patient and one untreated localized prostate cancer patient, but more CTCs were detected in the remaining eight cases by IsoFlux (**Fig 6A**) (**Table 4**).The mean number of harvested CTCs were 33.8 ± 28.3 and 37.6 ± 20.8 by Parsortix and IsoFlux respectively. There was no statistically significant difference (p = 0.33) between two systems. Both Parsortix and IsoFlux detected more than five CTCs in 90% (9/10) of samples. We also compared Parsortix and CellSearch (**Fig 6A**) (**Table 4**). Parsortix harvested significantly more (p = 0.04) CTCs and the mean numbers of harvested CTCs were 32.1 ± 29.1 and 10.1 ± 9.3 respectively. Parsortix harvested more CTCs in all the cases except PC22b. More than five CTCs were detected in 100% (7/7) of Parsortix harvested samples and in 43% (3/7) of CellSearch harvested samples. CTC purity was compared between IsoFlux and Parsortix isolated samples and Parsortix yielded a higher purity than IsoFlux in eight of the ten patients (**Fig 6B**). The mean purity of the Parsortix harvest at 3.1% ± 2.7% was significantly higher than that of IsoFlux at 1.0% ± 0.5% (p = 0.02), due to the far larger number of leucocytes in IsoFlux than Parsortix harvested cells.

### 5.The potential to harvest epithelial marker/EpCAM negative CTCs using Parsortix

**Fig 7:**
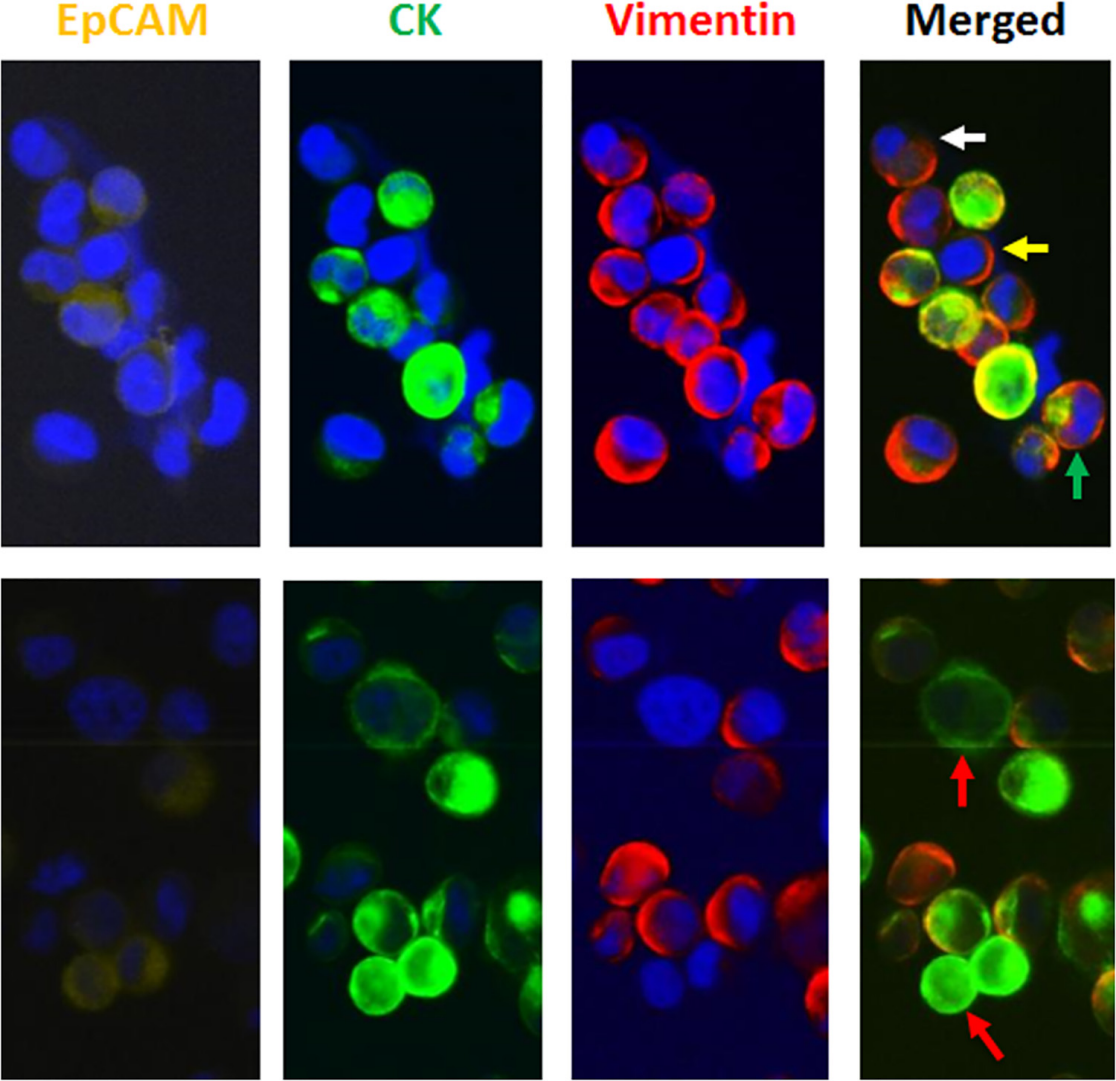
EMT expressions on PC3 cell lines. Various types of signals combination were presented including cells purely expressing epithelial markers without Vimentin (red arrow), purely expressing Vimentin without either EpCAM or CK (white arrow), expressing EpCAM but no CK (yellow arrow), and expressing CK but no EpCAM (green arrow).

PC3 cells were firstly used to test the existence of EMT in prostate cancer cells by EpCAM, CK and Vimentin immunostaining. Heterogeneous expression level of EpCAM, CK and Vimentin can be observed on those cells, with a small proportion (2.5%) of cells completely lacking the expression of epithelial markers. Moreover, 2.6% of cells expressed EpCAM but not CK and 8.7% of cells expressed CK but lacked EpCAM (**Table 5**)(**Fig 7**), indicating the existence of EMT, in particular epithelial negative EMT cells.

**Table 5:** The proportion of PC3 cells expressing epithelial and mesenchymal markers.

	EpCAM+/CK- number (%)	EpCAM+/CK+ number (%)	EpCAM-/CK+ number (%)	EpCAM-/CK- number (%)	Total number (%)
Vimentin+	18 (2.5%)	602 (83.3%)	61 (8.4%)	10 (1.4%)	691 (95.6%)
Vimentin-	1 (0.1%)	21 (2.9%)	2 (0.3%)	8 (1.1%)	32 (4.4%)
Total					723 (100.0%)

**Fig 8:**
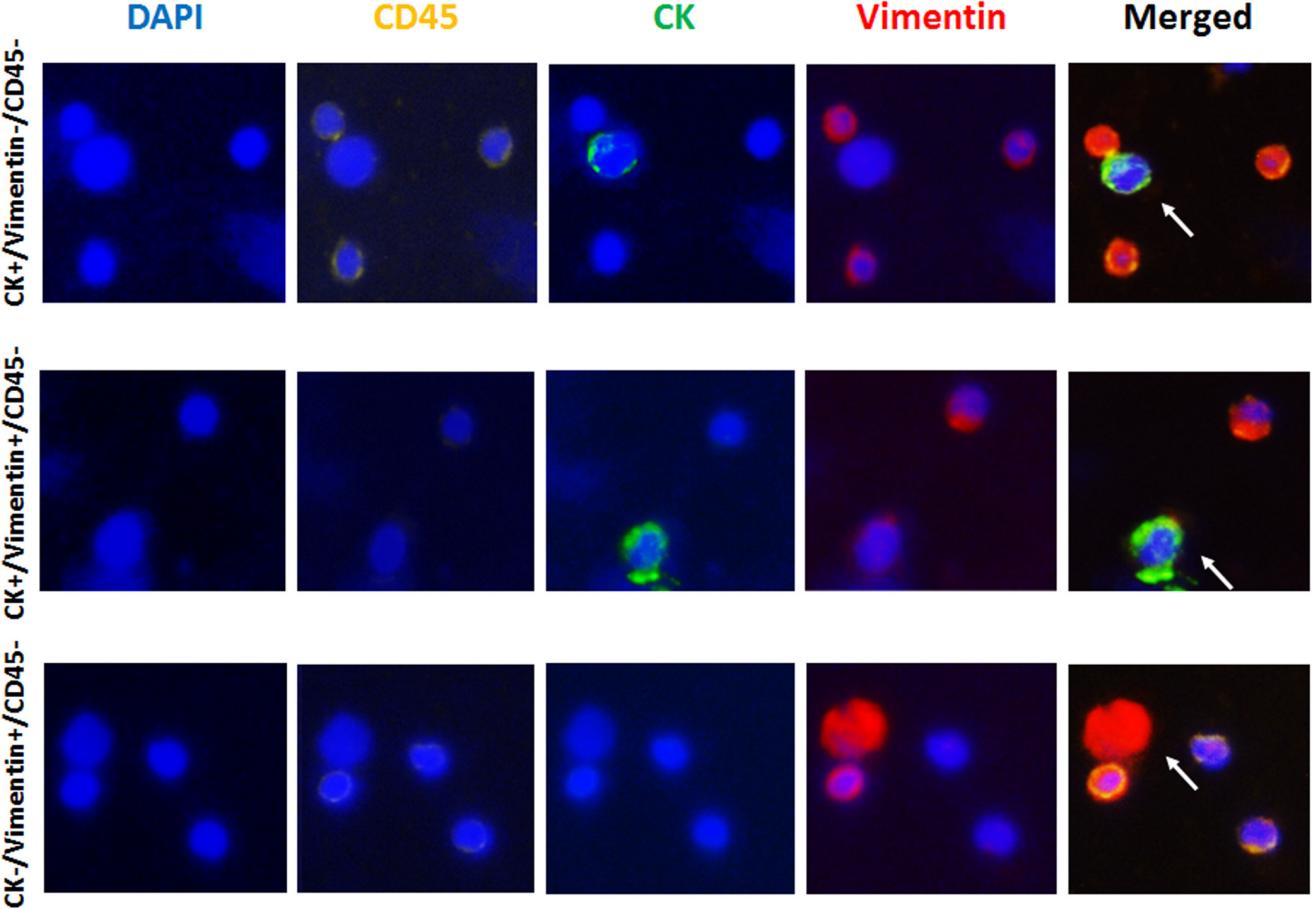
Representative images for different types of detected cells in prostate cancer patients. (A) A CK positive/Vimentin negative/CD45 negative cell surrounded by CD45 positive lymphocytes. (B) A CK positive/Vimentin positive/CD45 negative cell next to a CD45 positive lymphocyte. (C) A CK negative/Vimentin positive/CD45 negative cell surrounded by CD45 positive lymphocytes.

Using 4-color immunofluorescence, different populations of CK-positive/Vimentin-negative /CD45-negative, CK-positive/Vimentin-positive/CD45-negative, and CK-negative/Vimentin-positive/CD45-negative cells were observed and recorded for each patient (**Table 6**). Vimentin positive/CD45 negative cells were detected in four of five cancer patient samples used for this analysis and 0%, 15.7%, 27.8%, 71.4% and 92.9% of CK positive CTCs expressed Vimentin in these five patients, including three metastatic prostate cancer patients in treatment and two newly diagnosed untreated prostate cancer patients (**Tables 1 and 6**). Samples from three healthy donor controls showed no CK-positive/CD45-negative cells, while 0, 2 and 3 CK-negative/Vimentin-positive/CD45-negative cells were detected. Representative images are shown in **Fig 8**.

**Table 6:** CTC analysis data of clinical samples from prostate cancer patients by four-color immunofluorescences.

Case ID	CK+/Vimentin-/CD45-	CK+/Vimentin+/CD45-	CK-/Vimentin+/CD45-
PC32b	97	18	34
PC36	13	5	8
PC39	2	5	2
PC40	2	26	8
PC46	4	0	0

### 6. Parsortix isolated tumor cells are viable for cell culture

**Fig 9:**
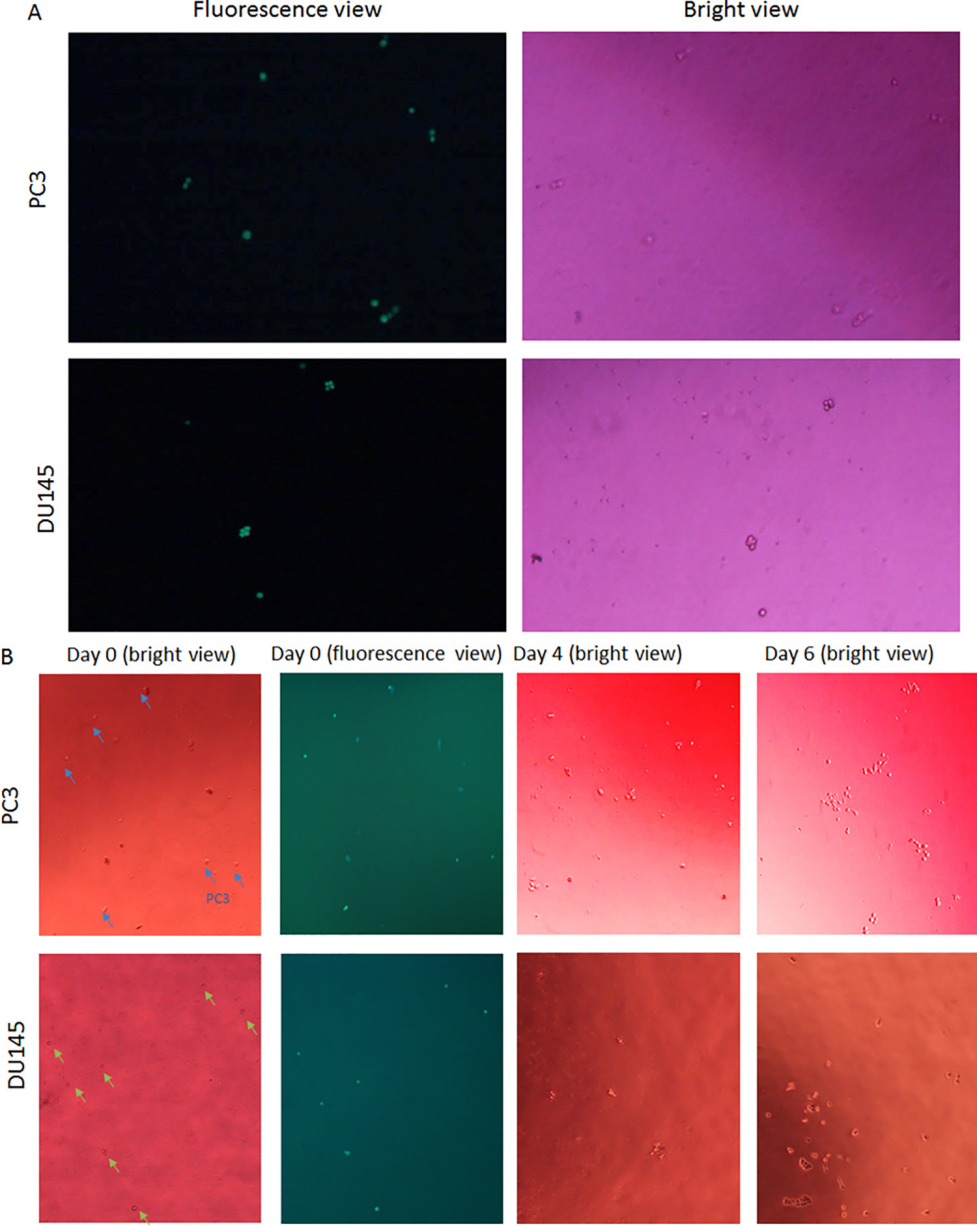
Viability of prostate cancer cell lines after isolation. (A) Pre-labeled cancer cell lines (labeled green) were identified with fluorescence microscope and then viewed in bright light to determine whether they were dyed with Trypan blue. Cells with clear cytoplasm were considered viable. (B) Cancer cells were cultured after isolation. Spiked pre-labeled cells (indicated by arrow) in 24-well plate, which were relative bigger and brighter comparing surrounding lymphocytes, under bright light and fluorescence views of the same area for spiked cells (labeled green) were present. Cancer cells attached to the plate bottom and started to proliferate on Day 4., They had grown well to form obvious clusters of proliferating cells by Day 6.

To determine if the processing of cells through Parsortix system will damage cells and affect their viability, we analyzed the viability of two prostate cancer cell lines (PC3 and DU145) after harvest by using trypan blue exclusion and cell proliferation analysis along with lymphocytes in culture. Trypan blue exclusion showed 100% of fluorescence-labeled cancer cells (either PC3 or DU145) were viable (**Fig 9A**). In culture dishes, pre-labeled cell lines surrounding by normal lymphocytes can be clearly identified under fluorescence microscope after harvesting into 24-well plate. On day 2, medium changing washed off most lymphocytes and cancer cells remained attached. Both cell lines grew into cell clusters on day 4 and formed obvious clusters on day 6 (**Fig 9B**).

## Discussions

Real-time monitoring of cancer genetic/molecular changes and consequent selection of the most-suited treatment based on the genetic alterations of tumor cells at the time when the treatment is being given to the patient will dramatically increase the treatment efficacy. It will also reduce healthcare costs in relation to ineffective drugs. Analysis of circulating biomarkers, requiring only a venous blood sample (a ‘‘liquid biopsy”), has the potential to be used for frequent real-time genetic/molecular analysis to guide tumor diagnosis/prognosis and therapy selection. CTCs hold much more genetic/molecular information than free circulating nucleic acid and proteins and can reveal inter-cell heterogeneity for cancer prognosis and treatment resistance correlation. Consequently, CTC analysis has the potential to become the main diagnostic tool for personalized treatment based on real-time acquired cellular and molecular features of the tumors. Many CTC isolation systems have been developed in recent years, and each has its advantages and limitations. In this study, we optimized and evaluated a size-based CTC harvesting platform, Parsortix.

The principle behind Parsortix CTC (cancer cell) isolation is that most cancer cells are much larger than peripheral blood lymphocytes, which has been demonstrated by morphometric analysis of several cancer cell lines derived from different organs, including the MCF-7 breast cancer and the PC3 and DU145 prostate cancer cell lines [20–24]. The major advantages of the size-based Parsortix system are its simple approach and operation, its epitope-independence and its relatively low cost consumables. It avoids the use of expensive antibodies required for the cell surface protein based isolation methods and also the low throughput for those size-based systems using a membrane to filter cells, which have been reported to be able to run only small quantities of whole blood samples [20] and with the problem that cells remain trapped in the filter membrane [20, 23, 25], limiting the downstream analysis. Easy recovery of captured cells out of the system means the Parsortix system has great advantage for its potential future utilization in clinical diagnosis/prognosis using CTCs.

At the time of this study, the Parsortix version 1 system was limited to running 3ml of whole blood with a relatively slow sample processing speed. Using larger volume blood samples will clearly capture more CTCs and the currently established diagnostic blood volume using the FDA approved CellSearch system is 7.5 mL. 1:1 dilution of blood samples in PBS increases the total volume of blood samples that can be run on this system to isolate CTCs, but the speed for CTC isolation is still slow, making it less efficient for clinical use. Pre-selection and re-suspension of nucleated cells (buffy-coat) in PBS has been used in many CTC isolation methods, but this preparation with 7.5 mL blood sample frequently clogs the system. By adding EDTA and BSA into the PBS buffer to prevent cell stickiness, we successfully developed an approach to significantly speed up CTC isolation, so that 7.5 ml or even bigger volume of blood sample can be run on the Parsortix within a short time and without affecting CTC harvest rate. The total CTC isolation time of two hours (40 min for buffy-coat recovery, 44 min for cell isolation and 45 min for automated rinse and CTC collection) for a 7.5 ml of blood sample, was quicker than IsoFlux (40 min for buffy-coat recovery, 2 h incubation of beads with cells and 45 min for cell isolation) and CellSearch (4 h in total for CTC isolation) systems. Therefore, this modification of sample preparation makes Parsortix version 1 efficient for clinical use, once CTC analysis isolated by Parsortix is proven to be of clinical benefit. While a version 2 Parsortix system is now available that processes 10 mL of whole blood in 2.5 h, the buffy coat method can still be combined with the Parsortix version 2 system to further accelerate CTC harvesting time when a number of clinical samples are required to be processed on one system on the same day.

Compared with CellSearch, IsoFlux and other cancer cell surface marker based antibody capture systems, Parsortix avoids the potential problem in downstream CTC analysis caused by the magnetic beads. For example, in immunofluorescence analysis, beads adhering to the cell membrane and covering on the top of the cells prohibits effective immunostaining for membrane proteins. Auto-fluorescence from the beads also affects CTC analysis using fluorescence markers, such as immunofluorescence and fluorescence *in situ* hybridization. The beads-free systems avoid these problems in downstream CTC analysis.

However, magnetic beads bound to cells have advantages in keeping cells on slides using a strong magnet during repeated washes for certain down-stream analyses such as immunofluorescence, which has been demonstrated by the IsoFlux system in harvesting almost all the EpCAM positive PC3 cell and retain them for immunofluorescence analysis. A large proportion (50% or more) cells collected from Parsortix system were lost during the process of transferring them onto the glass slide and down-stream immunofluorescence staining using the conventional 4% paraformaldehyde fixation method. Cytospin has been used by many researchers to improve cell transfer to the slide, but this method can only keep around 50% cells. Many cells were lost when they were transferred to the slide by the spin [26, 27]. In addition, the force to push cells onto the slide severely affects the cell morphology, which has been observed in our study and previously by other researchers [28]. Through a series of tests for different fixation methods and cell transfer solutions, we have developed a novel cell transfer and fixation method, which can keep more than 90% of cells after immunofluorescence staining.

We also compared the immunofluorescence signal after different fixation. Paraformaldehyde fixation is a standard method for immunostaining and generally preserves the cell morphology well. However, it is usually used for those cells growing on the slide and some samples are prone to fixative-induced auto-fluorescence [29]. In our study, while it provided good signals for immunofluorescence staining, a large proportion of cells were washed off during the staining process. Methanol can precipitate proteins, so that the protein “shell” of the cell is maintained, but certain antigenic epitopes can be completely destroyed by methanol, which is consistent with our data. Acetone usually permeabilizes well and is less damaging to cells. Therefore, in this study, acetone fixation not only kept almost all cells but also generated good signals for both membrane and cytoskeletal proteins.

When a large volume of isotonic buffer saline was used to re-suspend cells for air drying on a slide, a thick layer of salt was formed, causing cell loss after the cells were rehydrated. We attempted to reduce the amount of salt by drying cells in hypotonic solution, which resulted in a stable and acceptable rate of retained cells. Finally, we developed a novel procedure to harvest cells in hypotonic solution and fix cells using acetone. One potential problem of acetone fixed cells is the effect of cell permeabilization. In this study, we demonstrated that the cell surface protein CD45 and EpCAM always showed good IF signals after acetone fixation. Consequently, we have optimized an efficient method to transfer and fix cells on slide, which are suitable for immunostaining analysis not only for CTCs isolated by Parsortix but also generally for CTCs isolated by any systems and other rare cell samples. Intra-tumor heterogeneity is well recognized. Such improvement to transfer cells on to slide for molecular investigation is therefore important for the analysis of small number of cancer cells, such as CTCs or other rare cancer cells in body fluid, as losing few cells may lead to the missing of a subpopulation of cancer cells to be analyzed.

IsoFlux is a platform that uses magnetic beads to target cells and a microfluidic device containing an isolation zone to capture CTCs. It is reported to capture double the amount of CTCs in either spiked cell lines or matched prostate cancer samples compared to CellSearch [14]. Our study confirms that the IsoFlux system harvests many more cancer cells with epithelial features than CellSearch using EpCAM antibodies. Our PC3 EpCAM expression and spiking study showed that IsoFlux harvested almost all the EpCAM positive cells from the spiked blood sample. Comparing the efficiency of Parsortix with IsoFlux and CellSearch systems for spiking experiments using epithelial cancer cells, the capture rates in cassette (55%) and harvest rates on slide (43%) by Parsortix for the EpCAM positive PC3 cells were comparable to reported recovery rates by CellSearch (40%) but lower than those 80% as previous reported [14] and 90% in our data by IsoFlux. However, in patient samples, we demonstrated that Parsortix harvested more CK positive /CD45 negative CTCs than CellSearch and similar numbers to IsoFlux. Surprisingly, we used CK to identify more CTCs by Parsortix than by IsoFlux in two samples. Since the CTCs captured by IsoFlux are based on EpCAM expression, the capturing of more epithelial-like CTCs by Parsortix than IsoFlux may be explained by the fact that the expressions of EpCAM and CK are not always correlated. Some cells expressing high level of CK do not express EpCAM, and vice versa [30]. Our four-color immunofluorescence for PC3 cells also proved this phenomenon. A high proportion of CK positive but EpCAM negative CTCs might be an explanation why Parsortix harvested similar number of CTCs compared to IsoFlux and more than CellSearch, while for spiked PC3 it harvested less cancer cells than IsoFlux and similar number of cancer cells compared to CellSearch. This highlights the problem with EpCAM based antibody capture system for CTC isolation.

Although CellSearch has been approved by FDA for its clinical use in CTC analysis and IsoFlux showed much better harvest rate and purity than CellSearch for CTC isolation using EpCAM antibody, the major disadvantage of these immunomagnetic bead based systems is their dependence on cell membrane-expressed proteins [31, 32]. While other antibodies can be added to EpCAM antibody to make a cocktail for increased CTC capture rate, including EMT cells, an ideal cell surface biomarker to distinguish cancer cells from normal mononuclear cells is not available. Currently, CTC isolation is still mainly based on EpCAM expression on epithelial origin cancer cells [6]. Most importantly, EMT is more and more recognized to play an important role in metastasis and certain EMT cancer cells will lose EpCAM expression [16]. Isolation by techniques that are independent of marker expression, such as Parsortix, may help to capture those EMT CTCs. CTC clusters have been reported to have increased metastatic potential, be more resistant to apoptosis and be correlated with poorer prognosis compared to single CTCs [33]. In Parsortix isolated samples, we also observed clusters of more than three CTCs. The ability to obtain CTC clusters will help to understand the metastasis progenitor and to predict patient prognosis.

The current widely applied set of criteria to identify CTCs has its limitations, as we showed that in PC3 cells, 3.9% cells were negative for CK but positive for Vimentin. Using four-color immunofluorescence, we detected CK-positive/Vimentin-positive/CD45-negative and CK-negative/Vimentin-positive/CD45-negative cell population aside from CK-positive/Vimentin-negative/CD45-negative cells, and in some cases, particularly the high Gleason score ones, there were many Vimentin-positive/CD45-negative cells. Those CK and Vimentin double positive cells may be CTCs with a certain degree of EMT. However, it is difficult to be certain that those CK-negative/Vimentin-positive/CD45-negative cells are CTCs, as endothelial and fibroblast cells with the same protein expression pattern have been found in circulation [34, 35]. The much lower number of this type of cells in blood samples from healthy donors than the cancer patents indicates that at least a proportion of those CK-negative/Vimentin-positive/CD45-negative cells harvested by Parsortix may be mesenchymal CTCs. A large cohort study with clinical data is necessary to determine the extent to which the number of CK-negative/Vimentin-positive/CD45-negative cells is correlated with clinical features and/or outcomes.

Successful culture of CTCs isolated from cancer patients is of high interest, although the difficulty is also foreseeable. If CTCs can be cultured, sufficient cells will be generated for much more cellular and molecular feature investigation and CTCs can also be used to test the tumor response to therapies. Some researchers have successfully cultured CTCs *in vitro* and utilized them for study patterns of drug susceptibility and genetic alteration, but cases are very few [36–38]. Therefore, cell viability is another important factor to evaluate a CTC isolation system. Using prostate cancer cell lines to spike blood samples, we demonstrated that cells harvested from the Parsortix system are viable. As a small number of cells are usually difficult to culture, we spiked 200 cancer cells with expectation to harvest around 100 of them, mimicking the CTC numbers in certain patients with high number of CTCs and demonstrated that such a small number of cells processed through the Parsortix system with blood samples can be cultured to generate more cells. Clearly, here we only demonstrated that cancer cells are viable after Parsortix isolation. Culturing CTCs requires specific optimized culture medium and conditions and will remain a big challenge for CTC study.

A clear limitation of Parsortix and other cell size-based systems is the difficulty to completely separate cancer cells and leukocytes by their size. The size of the captured tumor cells from non-sized-based systems was reported to range from 4 to 30 μm [39]. The diameter of lymphocytes has been reported to be 7.1 to 10.5 μm using flow cytometry [21] and 5.2 to 10.1 μm using light microscopy of cell suspensions [22], which is similar to our results (a mean diameter of 7.3 μm, ranging from 5 to 14.7 μm, with a rare proportion of cells >10 μM). Size overlapping between CTCs and leukocytes will result in the loss of small CTCs and retention of certain leukocyte contamination. Parsortix uses 10 μm as the gap for CTC isolation, balancing the recovery rate and purity. Using two prostate cancer and one breast cancer cell lines to spike blood samples, we showed that 44–60% cancer cells can be captured by Parsortix while keeping around 1000 other cells in the harvest sample. One recent reported filter-based device using 8 μm-sized pore described a recovery rate of more than 85%, but the number of contaminated lymphocytes in the final sample was not mentioned [23]. As CTCs are rare, a large number of contaminated lymphocytes potentially make downstream CTC analysis very difficult, such as fluorescence *in situ* hybridization and next generation sequencing. This is also the reason that limits the application of density based approaches alone. For example, OncoQuick provides a high recovery rate of 87%, but an expected poor purity with around 9.5x10^4^ white blood cells after enrichment [24]. Each current CTC isolation platform has its limitation either with recovery rate, purity or throughput and most of them with more than one limitation [6]. As long as the system can isolate a proportion of CTCs and more importantly, information from those CTCs can be correlated with clinical prognosis or treatment response, it may be a useful CTC isolation system, suitable for clinical diagnosis. If the clinical value of CTCs isolated by Parsortix can be confirmed, Parsortix will make CTC analysis much easier for clinical diagnostic application owing to its simple approach to the capture and harvest of varying phenotypes of CTCs (epithelial, mesenchymal and clusters) with shorter isolation time, higher purity and without contaminating beads.

In summary, we evaluated and optimized Parsortix, a novel CTC isolation platform based on cell size. By modification of the sample preparation and isolated cell harvest and fixation methods, the system was suitable for processing blood samples with at least comparable speed and capacity of blood volume to the FDA approved CellSearch system. Captured cells can be efficiently harvested on to slides for immunofluorescence analysis by directly loading, air drying in hypotonic solution and fixing with acetone. In matched patient samples, Parsortix captured a similar number of CTCs to IsoFlux but with a higher purity and both these systems captured more CTCs than CellSearch. Using four-color immunofluorescence staining, mesenchymal markers can be detected on CD45 negative cells isolated by Parsortix, indicating the potential to capture CTCs with mesenchymal features. We also demonstrated that cells isolated by Parsortix were viable for culture. This study with the sample process optimization, demonstrated that the size-based Parsortix system has great potential for future application in CTC harvesting and downstream analysis.

## Supporting Information

S1 Table Diameter measurement for cancer cell lines and normal human lymphocytes. (DOCX)

S2 Table Number of all harvested cells after isolation by Parsortix and IsoFlux in matched clinical samples. (DOCX)
