# Supplementary material for: Optimization and Evaluation of a Novel Size Based Circulating Tumor Cell Isolation System
Source: PLoS One. 2015 Sep 23;10(9):e0138032. doi: 10.1371/journal.pone.0138032 (PMC4580600; doi:10.1371/journal.pone.0138032)
Supplement: S1 Table — (DOCX) [file pone.0138032.s001.docx]

S1 Table. Diameter measurement for cancer cell lines and normal human lymphocytes

|  | Mean Diameter (µm) | | |
| --- | --- | --- | --- |
| Type of cells | Test 1 | Test 2 | Test 3 |
| PC3 | 19.67±4.14 | 18.11±3.60 | 18.65±3.72 |
| DU145 | 17.68±3.78 | 15.84±3.54 | 16.76±3.66 |
| MCF-7 | 14.55±3.31 | 14.54±3.08 | 14.06±3.17 |
| Normal lymphocytes | 6.98±1.87 | 7.45±2.01 | 7.43±1.94 |
