# Supplementary material for: Optimization and Evaluation of a Novel Size Based Circulating Tumor Cell Isolation System
Source: PLoS One. 2015 Sep 23;10(9):e0138032. doi: 10.1371/journal.pone.0138032 (PMC4580600; doi:10.1371/journal.pone.0138032)
Supplement: S2 Table — (DOCX) [file pone.0138032.s002.docx]

S2 Table. Number of all harvested cells after isolation by Parsortix and IsoFlux in matched clinical samples

| Case ID | Parsortix | | IsoFlux | |
| --- | --- | --- | --- | --- |
|  | CTCs (n) | Other cells (n) | CTCs (n) | Other cells (n) |
| PC2 | 39 | 1140 | 50 | 3343 |
| PC5 | 94 | 990 | 36 | 5238 |
| PC15b | 19 | 743 | 22 | 2731 |
| PC16 | 54 | 746 | 64 | 10000 |
| PC17 | 7 | 1131 | 27 | 2434 |
| PC19 | 8 | 1008 | 33 | 2689 |
| PC7b | 23 | 1234 | 36 | 3321 |
| PC20 | 38 | 1014 | 76 | 3673 |
| PC28 | 2 | 853 | 5 | 3181 |
| PC37 | 54 | 1654 | 27 | 2085 |
